# Association between use of lung-protective ventilation with lower tidal volumes and risk of acute lung injury, mortality, pulmonary infection, and atelectasis: a meta-analysis

**DOI:** 10.1186/cc12654

**Published:** 2013-06-19

**Authors:** VGM Pereira, A Serpa Neto, SO Cardoso, JA Manetta, DC Espósito, M de Oliveira Prado Pasqualucci, MJ Schultz

**Affiliations:** 1 ABC Medical School (FMABC), Príncipe de Gales, Santo André, SP, Brazil

## Introduction

Lung-protective mechanical ventilation with the use of lower tidal volumes has been found to improve the outcome of patients with acute lung injury (ALI) or its more severe form acute respiratory distress syndrome. It has been suggested that use of lower tidal volumes also benefits patients not suffering from ALI. The objective of this study was to test the hypothesis that use of lower tidal volumes is associated with improved outcomes of patients without ALI.

## Methods

*Data source *A search of MEDLINE, CINAHL, Web of Science, and Cochrane Central Register of Controlled Trials up to August 2012. *Study selection *Eligible studies were those evaluating use of lower versus higher tidal volumes in patients without ALI at onset of mechanical ventilation and reporting lung injury development, overall mortality, pulmonary infection, atelectasis and biochemical alterations. *Data extraction *Three reviewers extracted data on study characteristics, methods, and outcomes. Disagreement was resolved by consensus.

## Results

Twenty articles (2,822 participants) were included. Metaanalysis using a fixed-effect model showed a decrease in lung injury development (risk ratio (RR), 0.33 (95% CI 0.23 to 0.47); number needed to treat (NNT), 1 to 11), mortality (RR, 0.64 (95% CI 0.46 to 0.89); NNT, 1 to 23) and pulmonary infection (RR, 0.52 (95% CI 0.33 to 0.82); NNT, 1 to 26) in patients ventilated with lower tidal volumes. The results of lung injury development were similar when stratified by the type of study (randomized vs. nonrandomized), was significant only in randomized trials for pulmonary infection and only in nonrandomized trials for mortality. The hospital length of stay was lower in the protective ventilation group (6.91 ± 2.36 vs. 8.87 ± 2.93 days, respectively; standardized mean difference, 0.60 (95% CI 0.50 to 0.71)). Protective ventilation was associated with higher PaCO_2 _levels (41.05 ± 3.79 vs. 37.90 ± 4.19 mmHg, respectively; SMD, -0.47 (95% CI -0.59 to -0.34)), lower pH (7.37 ± 0.03 vs. 7.40 ± 0.04, respectively; SMD, 0.75 (95% CI 0.58 to 0.92)) but similar PaO_2_/FiO_2 _(304.40 ± 65.7 vs. 312.97 ± 68.13, respectively; SMD, 0.08 (95% CI 0.00 to 0.16)). The tidal volume gradient between the two arms did not influence significantly the final results.

## Conclusion

Among patients without ALI, protective ventilation with lower tidal volumes was associated with a better clinical outcome. Some of the limitations of our meta-analysis were the mixed setting of mechanical ventilation (ICU or operating room) and the duration of mechanical ventilation.

